# Sending the Signal to Bone: How Tumor-Derived EVs Orchestrate Pre-Metastatic Niche Formation and Skeletal Colonization

**DOI:** 10.3390/biomedicines13071640

**Published:** 2025-07-04

**Authors:** Alhomam Dabaliz, Hagar Mahmoud, Raffi AlMutawa, Khalid S. Mohammad

**Affiliations:** 1 Department of Clinical Skills, College of Medicine, Alfaisal University, Riyadh 11533, Saudi Arabia; almdabaliz@alfaisal.edu; 2 Department of Anatomy, College of Medicine, Alfaisal University, Riyadh 11533, Saudi Arabia; hmmahmoud@alfaisal.edu (H.M.); raalmutawa@alfaisal.edu (R.A.)

**Keywords:** extracellular vesicles, bone metastasis, pre-metastatic niche, osteoclastogenesis, liquid biopsy biomarkers, EV-targeted therapy

## Abstract

Bone is a preferred site for disseminated tumor cells, yet the molecular mechanisms that prepare the skeletal microenvironment for metastatic colonization are only beginning to be understood. At the heart of this process are extracellular vesicles (EVs), nano-sized, lipid-encapsulated particles secreted by cancer cells and stromal components. This review consolidates current findings that position EVs as key architects of the bone-metastatic niche. We detail the biogenesis of EVs and their organotropic distribution, focusing on how integrin patterns and bone-specific ligands guide vesicle homing to mineralized tissues. We then outline the sequential establishment of the pre-metastatic niche, driven by EV-mediated processes including fibronectin deposition, stromal cell reprogramming, angiogenesis, neurogenesis, metabolic reconfiguration, and immune modulation, specifically, the expansion of myeloid-derived suppressor cells and impaired lymphocyte function. Within the bone microenvironment, tumor-derived EVs carrying microRNAs and proteins shift the balance toward osteoclastogenesis, inhibit osteoblast differentiation, and disrupt osteocyte signaling. These alterations promote osteolytic destruction or aberrant bone formation depending on tumor type. We also highlight cutting-edge imaging modalities and single-EV omics technologies that resolve EV heterogeneity and identify potential biomarkers detectable in plasma and urine. Finally, we explore therapeutic approaches targeting EVs, such as inhibition of nSMase2 or Rab27A, extracorporeal EV clearance, and delivery of engineered, bone-targeted vesicles, while addressing translational challenges and regulatory considerations. This review offers a roadmap for leveraging EV biology in predicting, preventing, and treating skeletal metastases by integrating advances across basic biology, bioengineering, and translational science.

## 1. Introduction

Bone metastasis is a common and serious complication of advanced malignancies, particularly in patients with lung, breast, and prostate cancers [1], as well as those with multiple myeloma [2]. Epidemiological studies show that among individuals with metastatic disease, bone metastases are present in approximately 88.7% of prostate cancer cases, 53.7% of breast cancer cases, and 38.7% of renal cancer cases [3]. In the United States alone, it is estimated that 300,000 to 600,000 individuals live with metastatic bone disease (MBD) each year [4,5,6]. Notably, the 5-year cumulative incidence rates of bone metastases reach 52% for prostate cancer, 41% for breast cancer, and 33% for lung cancer [6].

The clinical burden of bone metastases is significant, primarily due to the development of skeletal-related events such as pathological fractures, spinal cord compression, and the need for surgical or radiotherapeutic interventions [7,8]. These complications diminish patients’ quality of life, resulting in substantial healthcare costs. For example, the annual cost attributable to skeletal metastases is estimated at around USD 18,272 per patient. Moreover, the average direct medical cost for patients with MBD is approximately USD 75,329, compared to USD 31,382 for patients without bone involvement [9]. Overall, the financial impact of bone metastases in the United States exceeds USD 12.6 billion annually, accounting for approximately 17% of total cancer care expenditures [9].

**Figure 1:**
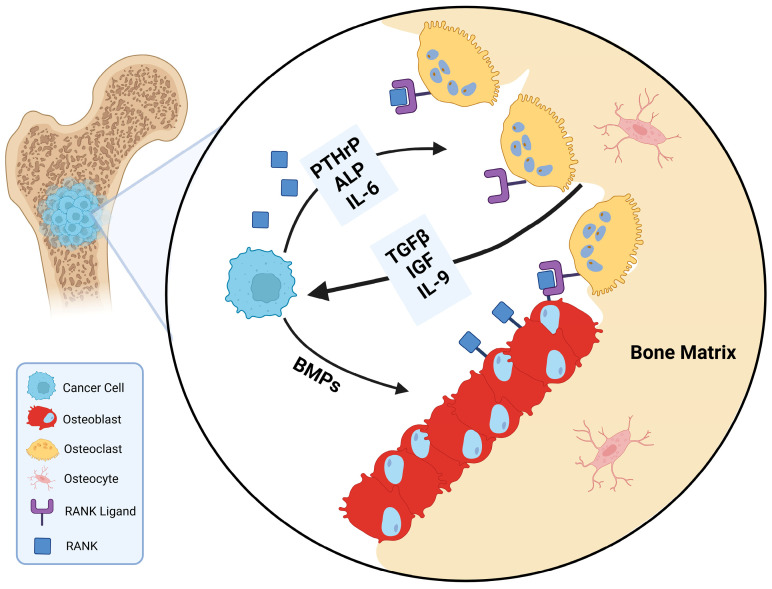
The “vicious cycle” of bone metastasis. Disseminated tumor cells that localize to bone initiate a destructive feed-forward loop by secreting osteolytic mediators, mainly parathyroid hormone-related protein (PTHrP), which stimulates osteoblasts to increase expression of receptor activator of nuclear factor-κB ligand (RANKL). RANKL engages its receptor RANK on osteoclasts, promoting their activity. Osteoclast-mediated degradation of the bone matrix releases a reservoir of growth factors, including transforming growth factor-β (TGF-β), insulin-like growth factors (IGFs), and bone morphogenetic proteins (BMPs), which in turn enhance tumor cell proliferation and further PTHrP production. Alkaline phosphatase (ALP) activity in osteoblasts indicates reactive bone formation at the metastatic interface, while osteocytes contribute to the amplification of osteoclastogenic and pro-tumorigenic signaling through interleukins 6 and 9. Collectively, these reciprocal interactions establish a self-sustaining loop in which skeletal degradation and tumor expansion are mutually reinforcing, underscoring the therapeutic imperative to concurrently target tumor cells, RANKL-driven osteoclast activation, and the release of bone-derived growth factors. Created in BioRender. Mohammad, K. (2025) https://BioRender.com/rojtj52.

In terms of underlying mechanisms, the progression of bone metastasis has long been described by the “vicious cycle” model, first proposed in the late 1990s [10]. This cycle is initiated when the metastasized cancer cells secrete osteolytic factors like parathyroid hormone-related protein (PTHrP) and Jagged 1. This process stimulates osteoclastic bone resorption, releasing tumor-growth factor β (TGF-β) from the mineralized bone matrix, further promoting osteolytic factor release. Jagged 1 also induces the differentiation of monocytes to osteoclasts, while PTHrP increases osteoblastic production of receptor activator of nuclear factor κB ligand (RANKL), a major driver of osteoclastogenesis (Figure 1). These newly differentiated osteoclasts will further degrade the bone matrix, releasing more of these osteolytic factors, and fueling this feedforward cycle further [11,12]. While this framework provides a strong foundation for understanding bone metastasis, it does not fully capture the complexity of tumor–bone interactions.

Recent advances have revealed that extracellular vesicles (EVs), small, membrane-bound particles secreted by tumor cells, play a crucial role in intercellular communication within the bone microenvironment [13,14]. For example, EVs secreted from osteoclasts contain cargo that inhibits osteoblastic activity and promotes osteoclastogenesis, while osteoblast-derived EVs carry cargo that plays a central role in inducing osteoblast differentiation [13]. Bone marrow stem cells also induce osteogenic differentiation of mesenchymal stem cells through EVs [13]. However, just as normal cells can exert their effects through EVs, tumor-derived EVs (TDEVs) can deliver a variety of bioactive molecules that modulate the activity of many cells, such as osteoblasts and osteoclasts, to support the formation of pre-metastatic niches, and contribute to tumor progression and resistance to therapy [15,16,17]. Incorporating EV-mediated signaling into the “vicious cycle” model offers a more comprehensive understanding of the molecular mechanisms driving bone metastasis and may uncover novel therapeutic targets [18].

## 2. Biological Foundations of EVs and the Bone Microenvironment

### 2.1. EV Biogenesis, Cargo, and Organotropism

Extracellular vesicles (EVs) are lipid bilayer-enclosed particles secreted by virtually all cell types, playing a central role in intercellular communication by transferring proteins, lipids, and RNAs [19]. Based on their biogenesis and size, EVs are broadly classified into three main categories: exosomes (30–150 nm), which originate within multivesicular bodies (MVBs) [20,21]; microvesicles (100–1000 nm), which bud directly from the plasma membrane [22]; and apoptotic bodies (500–2000 nm), formed during the late stages of programmed cell death [23]. Understanding these distinct biogenetic pathways is fundamental, as the structural and molecular features of EVs directly influence their functional roles in both physiological and pathological contexts [24].

EVs have gained significant attention for their contribution to cancer, where they facilitate tumor progression and metastasis by reshaping distant microenvironments [13,25]. Exosomes, a major subtype, encapsulate molecular cargo that reflects the identity of their originating cells [26], enabling them to modulate immune responses [27], support tissue repair, and drive malignancy through the horizontal transfer of oncogenic factors [28]. In parallel, microvesicles actively participate in intercellular signaling by delivering proteins and RNAs involved in critical biological processes such as coagulation, inflammation, and cellular migration [29,30]. In the context of bone malignancies like osteosarcoma, tumor-derived EVs (TDEVs) promote tumor growth, angiogenesis, immune suppression, metastasis, and chemoresistance by reprogramming the bone microenvironment [31]. Similarly, in bone metastases from breast, prostate, and lung cancers, EVs critically alter the bone marrow niche by inducing stromal remodeling, enhancing osteolysis, and modulating immune activity, thereby establishing a pre-metastatic landscape favorable for tumor colonization and disease progression [32,33].

EVs, mainly exosomes, are produced through tightly regulated biogenesis pathways that primarily involve the Endosomal Sorting Complex Required for Transport (ESCRT) machinery [34,35,36,37]. This system orchestrates the budding and scission of intraluminal vesicles (ILVs) within multivesicular bodies (MVBs) via a sequential process involving cargo recognition by ESCRT-0, membrane budding initiated by ESCRT-I and ESCRT-II, and membrane scission mediated by ESCRT-III and VPS4 [38,39]. Upon maturation, MVBs fuse with the plasma membrane, releasing ILVs as exosomes loaded with selectively sorted bioactive cargo that mirrors the molecular characteristics of their parent cells [34,35,36,37].

In addition to ESCRT-dependent pathways, EV biogenesis can proceed via ESCRT-independent mechanisms. In these routes, the bioactive lipid ceramide, generated by neutral sphingomyelinase 2 (nSMase2), induces negative membrane curvature to facilitate vesicle budding, while tetraspanin-enriched microdomains such as CD9, CD63, and CD81 cluster specific cargo and regulate membrane dynamics independently of the ESCRT machinery [40,41,42]. These complementary pathways underscore the remarkable plasticity of EV production across a range of physiological and pathological conditions.

In EVs, lipids constitute the limiting bilayer that defines vesicle shape, stability, and biological activity [43]. Tumor cells, especially under hypoxic stress, reprogram lipid metabolism by enhancing de novo lipogenesis, lipid droplet formation, and fatty acid β-oxidation to support survival and growth in oxygen-deprived environments [44]. This metabolic rewiring directly influences EV biology, as lipidomic analyses reveal that highly metastatic breast cancer cells selectively package distinct phospholipids and sphingolipids into their EVs, a pattern linked to their potential for skeletal metastasis [45]. Furthermore, tumor-specific lipid profiles correlate with EV biogenesis and uptake mechanisms across various cancer types [46].

Among lipids, cholesterol plays a pivotal role in EV formation and function. The activation of the TGF-β–MEK/ERK–SREBP2 signaling axis elevates intracellular cholesterol levels, triggering an EV secretion burst [47]. The oxysterol metabolite 27-hydroxycholesterol further promotes EV release by preventing multivesicular bodies’ degradation in lysosomes [48]. Cholesterol-enriched lipid rafts within EV membranes enhance their structural stability and facilitate uptake by recipient cells through raft-dependent endocytosis [49]. Experimentally, increasing cholesterol in triple-negative breast cancer cells induces a surge in EV release [50], whereas cholesterol depletion in bone marrow myeloid cells impairs EV uptake, reduces NF-κB signaling, and significantly diminishes bone metastatic burden in prostate cancer models [25].

Ceramide, generated by neutral sphingomyelinase-2 (nSMase2), is another critical lipid regulator of EV biogenesis. Ceramide facilitates membrane curvature and intraluminal budding within multivesicular bodies, essential steps for EV formation. Genetic or pharmacological inhibition of nSMase2 decreases EV secretion and suppresses osteoclast activation and tumor cell motility [41,51]. The nSMase2 inhibitor GW4869 and related compounds thus represent promising therapeutic candidates to curb metastasis [52].

Arachidonic acid (AA) metabolism also plays a vital role in tumor progression and EV-mediated remodeling of the bone microenvironment. TDEVs enriched in enzymes such as COX-2, 12-LOX, and autotaxin (ATX) generate bioactive lipids, including prostaglandin E_2_ (PGE_2_), 12-hydroxyeicosatetraenoic acid (12-HETE), leukotriene B_4_ (LTB_4_), and lysophosphatidic acid (LPA), which promote osteoclastogenesis, bone pain, and matrix degradation [53,54,55]. Additionally, free arachidonic acid stimulates bone marrow adipocyte differentiation and enhances prostate cancer cell invasion through stromal barriers, effectively conditioning the bone niche for metastatic colonization [56].

Collectively, these dysregulated lipid pathways—cholesterol enrichment, ceramide production, and arachidonic acid metabolism—reprogram EV biogenesis, cargo sorting, and uptake, thereby sculpting a pro-osteolytic, immunosuppressive pre-metastatic niche in the bone.

**Figure 2:**
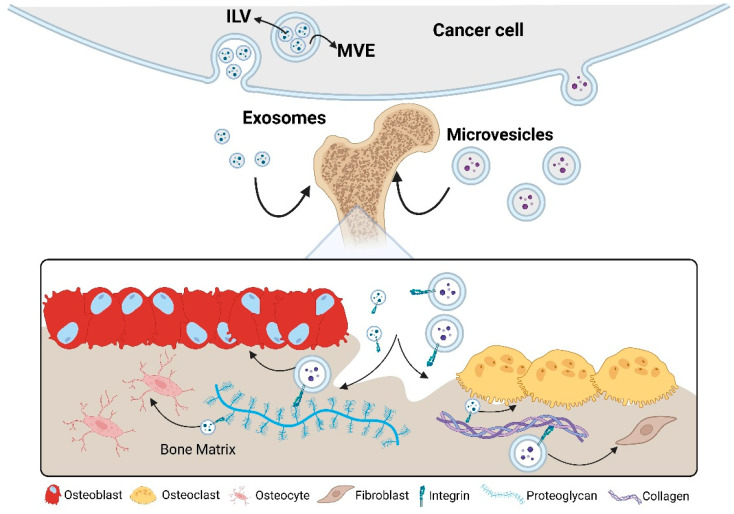
Biogenesis, bone-tropism, and cellular uptake of tumor-derived extracellular vesicles (EVs). The schematic illustrates (i) intraluminal vesicle (ILV) formation within multivesicular endosomes (MVEs) and subsequent release of exosomes (30–150 nm) alongside larger microvesicles (100–1000 nm) from the plasma membrane of a cancer cell; (ii) the molecular interface between circulating EVs and the mineralized bone matrix, highlighting integrin-mediated binding to collagen, proteoglycans, and other matrix ligands; and (iii) downstream uptake of EVs by osteoclasts, osteoblasts, osteocytes, and bone-resident fibroblasts, which re-program these cells toward a pro-metastatic phenotype. Key structural elements (lipid bilayer, integrins, representative cargo) and bone-matrix components are annotated for clarity. Created in BioRender. Mohammad, K. (2025) https://BioRender.com/kmzro5f.

EVs play a central role in cancer progression by mediating organotropic metastasis. This specificity is primarily influenced by the expression of integrins on the EV surface, which direct vesicle homing to particular tissues. For example, a recent study has shown that integrin α4 found on EVs of certain tumors facilitates selective capture by pancreatic and endothelial cells [57]. In another recent study, EVs expressing integrin α6 were found to localize to lung fibroblasts and epithelial cells; those expressing integrin β5 were found to localize to liver Kupffer cells, and those expressing integrin β3 localized to CD31+ endothelial cells within the brain [58]. When looking at the bone specifically, integrin αvβ3, commonly enriched on breast cancer TDEVs, facilitates adhesion to bone matrix proteins such as fibronectin and osteopontin, promoting metastatic colonization of the bone [59,60,61,62]. Similarly, integrin αvβ5 binds vitronectin and is implicated in the preferential dissemination of tumor cells to bone tissues [63]. Beyond their role in initial adhesion, EVs carrying integrin β3 actively remodel the bone microenvironment by promoting osteoclast differentiation and enhancing bone resorption, thereby establishing a pre-metastatic niche that supports tumor growth [32,64] (Figure 2).

### 2.2. Physiology of Bone Remodeling

Bone remodeling is a dynamic, tightly regulated process driven by the coordinated actions of osteoblasts, osteoclasts, and osteocytes. Osteoblasts, derived from mesenchymal stem cells, synthesize and mineralize bone matrix, whereas osteoclasts, originating from hematopoietic precursors, resorb bone by secreting acid and proteolytic enzymes; both activities are governed largely by the RANK/RANKL/OPG signaling axis [65]. Osteocytes, the most abundant and long-lived bone cells, are embedded within the mineralized matrix, act as mechanosensors, and secrete key regulators such as sclerostin, RANKL, and OPG [66]. They control remodeling through two major pathways: inhibition of Wnt/β-catenin signaling by sclerostin and modulation of osteoclastogenesis via the RANKL/OPG ratio. Sclerostin, encoded by the SOST gene, binds LRP5/6 receptors to suppress Wnt signaling and thus limits osteoblast-mediated bone formation [67].

The RANKL:OPG balance—primarily set by osteocytes and osteoblasts—determines osteoclast differentiation and is finely tuned by mechanical loading and hormonal cues [68,69]. Mechanical loading or intermittent parathyroid hormone (PTH) downregulates sclerostin, activate Wnt/β-catenin signaling, and stimulate bone formation, whereas unloading, continuous PTH, or pro-inflammatory cytokines increase sclerostin or RANKL, tipping the balance toward resorption [69,70]. Calcitonin further tempers resorption by directly inhibiting osteoclasts and may raise sclerostin levels, suppressing formation. Apoptotic osteocytes—often a consequence of disuse or microdamage—release ATP and other factors that upregulate RANKL in neighboring cells, triggering localized remodeling.

The disruption of these pathways underlies several diseases: osteoporosis (elevated RANKL or reduced OPG) [68]; sclerosteosis and Van Buchem disease (SOST mutations and sclerostin deficiency, leading to excessive bone formation) [67]; and inflammatory bone loss in conditions such as rheumatoid arthritis, where TNF-α and IL-6 enhance osteoclastogenesis. Growth factors in the TGF-β and BMP families are also essential for embryonic skeletal development and postnatal bone homeostasis [71,72,73].

### 2.3. Bone as a Privileged EV Target

The bone is a privileged target for EV uptake due to its unique anatomical and molecular characteristics that facilitate efficient EV delivery and retention. Bone’s extracellular matrix (ECM), characterized by its mineralized, porous structure enriched with collagen, hydroxyapatite, and glycoproteins, serves as a dynamic reservoir for EVs, facilitating their retention and function [74]. ECM-based biomaterials such as hydrogels and decellularized scaffolds further enhance EV localization and sustained release, positioning bone as a prime site for EV-mediated signaling and potential therapeutic applications [74].

Collagen, the most abundant ECM protein, provides structural integrity and a binding framework for EVs. Matrix vesicles (MVs), a subset of EVs secreted by osteoblasts, adhere to collagen fibrils and initiate mineral deposition by releasing calcium and phosphate ions, ultimately forming hydroxyapatite crystals within the collagen matrix. [75,76]. The ability of collagen to bind vesicles and direct them towards different cells in the bone environment exemplifies how ECM components actively participate in EV-mediated processes, promoting localized mineralization and targeted signaling [77].

The bone ECM is rich in integrins and proteoglycans, acting as docking sites for EVs and facilitating targeted binding to bone-resident cells. Integrins such as α5β1 and αvβ3 on EV surfaces interact with ECM proteins, enabling localized delivery of EV cargo and influencing osteogenesis and bone remodeling [78]. This selective adhesion mechanism is further demonstrated by αvβ3 integrin-mediated EV binding to ECM components. These ECM components transport these EVs to other cells within the bone tissue, assisting these recipient cells in taking up the EVs. Many studies have targeted these integrins as potential therapeutic targets for anti-cancer medications, and many studies have found a significant cancer-inhibitory effect when testing integrin blocking agents. [79]

The bone marrow vascular network is a highly specialized and dynamic system, comprising arterioles derived from nutrient arteries, transitional capillaries located near the endosteum, and fenestrated sinusoids that drain into a central venous sinus. This hierarchical architecture supports efficient molecular exchange, ensures adequate oxygen and nutrient delivery, and sustains both hematopoiesis and bone homeostasis [80,81,82]. In addition, it plays a key role in maintaining hematopoietic stem cell (HSC) quiescence and spatial organization within the perisinusoidal niche, while also facilitating the trafficking and distribution of EVs throughout the marrow microenvironment [83,84].

The vascular network’s proximity to bone cells enables efficient EV delivery to target cells, such as osteoblasts and osteocytes. Mesenchymal stem cell-derived EVs (MSC-EVs) are particularly significant in bone regeneration and homeostasis [85,86]. Under mechanical loading or injury, mechanically activated EVs (MA-EVs) enriched with angiogenic factors like VEGF are released, promoting endothelial cell proliferation and migration to coordinate angiogenesis with osteogenesis [87,88]. Additionally, MSC-EVs carrying key molecules like VEGF and HIF-1α further enhance vascularization, accelerating bone defect healing through targeted delivery to bone marrow cells [89,90].

Heparan sulfate proteoglycans (HSPGs), key components of the bone extracellular matrix (ECM), are essential regulators of osteogenesis and tissue remodeling. Growth factors such as VEGF and PDGF, along with cytokines like IL-8 and TGF-β, are frequently packaged into systemic EVs, especially TDEVs, and bind to HSPGs via specialized binding domains. Once delivered, these vesicle-associated signaling molecules are enzymatically released into the bone microenvironment through EV-associated heparanase and proteases, contributing to matrix remodeling and local cellular responses [91,92]. TDEVs are nanoscale vesicles secreted by tumor cells that transport a diverse cargo of bioactive molecules, including cytokines, growth factors, nucleic acids, and enzymes, and are critical mediators of metastatic niche preparation [93,94]. Through these mechanisms, HSPGs not only enhance the retention of TDEVs within bone tissue but also regulate the localized release of their metastatic cargo. This interaction contributes directly to the establishment of the pre-metastatic niche (PMN)—a permissive microenvironment shaped by primary tumors to support the survival, engraftment, and eventual outgrowth of disseminated tumor cells [58,95,96].

Experimental studies support the significance of the interactions between EVs and the ECM, showing that EVs are influenced by the ECM’s composition, with specific matrix-binding motifs enhancing EV uptake by bone cells. The RGD (Arg-Gly-Asp) motif is an example of one present in many ECM proteins, like fibronectin, and effectively facilitates the integrin-mediated binding of EVs [79]. Proteomic analyses of EV cargo have identified EV proteins involved in cell adhesion, differentiation, and ECM organization (for review, see [97]).

The structured nature of the bone ECM, combined with its diverse cellular environment, creates a unique microenvironment that retains EVs and actively participates in EV-mediated signaling. This dynamic interplay positions the bone as a privileged target for EVs [98].

## 3. EV-Driven Formation of the Pre-Metastatic Bone Niche

**Figure 3:**
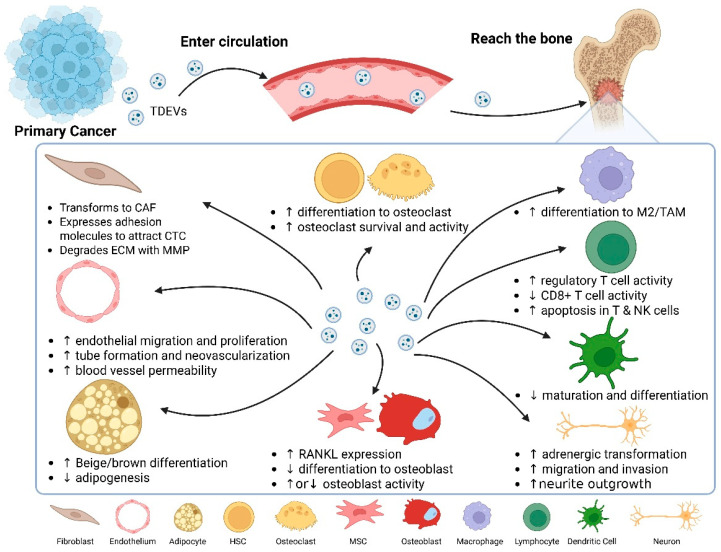
Systemic actions of tumor-derived EVs (TDEVs) during pre-metastatic niche formation in bone. Starting at the primary tumor, TDEVs enter the bloodstream and home to bone, where their cargo re-educates multiple stromal and immune populations. The diagram summarizes the principal functional effects detected to date: fibroblast activation to cancer-associated fibroblasts (CAFs) with matrix metalloproteinase (MMP) release; endothelial proliferation, tube formation, and vascular leakiness; adipocyte lipolysis and beige/brown trans-differentiation; skewing of hematopoietic stem cells (HSCs) toward osteoclastogenesis; modulation of osteoblast/osteoclast balance via altered RANKL expression; polarization of macrophages to M2/TAMs; suppression of cytotoxic lymphocytes and dendritic cell maturation; and neuro-adrenergic remodeling that favors tumor cell invasion. Arrows indicate the dissemination path (primary tumor → circulation → bone) and up- or downregulation (↑/↓) of each cellular process. Created in BioRender. Mohammad, K. (2025) https://BioRender.com/bobizhh.

Tumors can induce changes in the microenvironments of distant organs (Figure 3). These changes enhance the tumor cells’ ability to survive and grow in those distant organs once they arrive. These altered microenvironments have been termed “Pre-metastatic niches” (PMNs) [99,100,101]. Recent literature proposes that PMNs are primarily the result of the effects of tumor-secreted factors (TSFs) and tumor-derived extracellular vesicles (TDEVs) on the target organs [95].

The release of TDEVs is one of the first hallmarks of the tumor dissemination process [102]. These TDEVs, alongside tumor-secreted factors, cause a chain of events that form the PMNs at the target organ [103]. Among the primary effects of TDEVs on the microenvironment are stromal effects, such as the reprogramming of the fibroblasts in the PMN to produce inflammatory cytokines and growth factors such as Stromal Cell-Derived Factor 1 (SDF1) and TGF-β, which are both accelerators of tumor migration and invasion [104,105,106]. These PMN fibroblasts, known as cancer-associated fibroblasts (CAF), can also express fibronectin, an adhesion receptor that attracts circulating tumor cells (CTC) [107], and matrix metalloproteinases (MMPs), which enhance ECM degradation and tissue remodeling to promote CTC outgrowth [108]. Factors like SDF1 also recruit specific immune cells to the PMN, like CD11b+ myeloid cells, which express various integrins, inflammatory mediators, and angiogenic factors that all further promote the formation of the PMN [109]. CAFs can secrete EVs enriched with lysyl oxidase (LOX) as well, which contributes to PMN formation by crosslinking collagen fibers, increasing ECM stiffness, promoting CTC adhesion, and inducing the epithelial–mesenchymal transition [110,111,112]. This transition is primarily mediated by the FAK/paxillin/YAP pathway, initiated by the binding of the crosslinked collagen to integrin α2β1 in tumor cells. This binding phosphorylates the membrane-associated FAK/paxillin, activating them and ultimately leading to the nuclear translocation of the yes-associated protein (YAP), which promotes the epithelial–mesenchymal transition. All these factors make the ECM of the PMN a fertile location for CTC seeding.

TDEVs and TSFs can also induce an angiogenic shift in the PMN [113]. Tumor-secreted VEGF alongside recruited TIE2+ macrophages generate a proangiogenic microenvironment within the PMN [114]. Some tumors, like prostate and ovarian cancers, may secrete sphingomyelin and CD147 within TDEVs, which promote proangiogenic activity [115,116]. TDEVs may also contain many microRNAs (miRs) that promote angiogenesis, such as miR-9, miR-210, and miR-135, as well as miRs that increase the permeability of the microvasculature, thus promoting metastasis, such as miR-105 and miR-181c [117,118,119,120,121,122,123]. Another major contributor that has been identified within TDEVs of many cancers, including breast cancer and fibrosarcoma, is annexin A2 [124]. This calcium-dependent, phospholipid-binding protein has been implicated in promoting angiogenesis. This molecule achieves this by assisting in the conversion of plasminogen to plasmin [125], binding ECM molecules that regulate cell migration and proliferation of endothelial cells like tenascin C [126,127], and enhancing CTC adhesion, invasion, and proliferation [128,129,130].

Metabolic reprogramming is a major feature of malignant cells and the tumor microenvironment [131,132]. One of the most studied changes in these cells is the shift to anaerobic metabolism, like glycolysis and the Cori cycle [133]. Many factors, including hypoxia and specific molecules such as lysophosphatidic acid (LPA), have triggered this shift [134,135]. While these changes mainly occur in cancer cells, it is evident that the cancer microenvironment can be influenced by these cells to adopt similar metabolic habits [136]. However, it remains unclear whether these metabolic changes also happen in the PMN before metastatic seeding, and whether TDEVs would have a role in this change.

As the study of TDEVs’ effects on PMNs has garnered increasing attention, the search for a noninvasive way to track these TDEVs in vivo has intensified [137]. Recent advancements have identified a few exciting developments, notably labeling with DiR or Gaussia luciferase (gLuc). DiR is a lipophilic dye that exhibits a strong fluorescence signal when incorporated into a lipid membrane, and does not affect the vesicle’s morphology or integrity [138,139,140]. On the other hand, gLuc is a reporter protein that luminesces in response to coelenterazine, its substrate, by fusing with lactadherin, an EV-tropic protein [141,142]. Studies using labeled TDEVs have shown preferential accumulation in specific organs like the lung, liver, and bones, depending on the primary source of these vesicles, and continue to assist us in studying the effects of TDEVs on the target organ microenvironments [143,144].

## 4. Tumor EV-Mediated Dysregulation of Bone Remodeling Cells

The effects of TDEVs on the bone microenvironment extend even to the main cytotypes of bone tissue: osteoblasts, osteoclasts, and osteocytes (Table 1). Many cancers that metastasize to the bone, like breast cancer, lung cancer, and multiple myeloma, have been shown to interact extensively with these cells to create a fertile environment for the CTC seeding [145,146,147,148].

### 4.1. Osteoclastogenesis and Hyper-Resorptive/Osteolytic Phenotypes

Most bone metastasis presents in an osteolytic phenotype [184]. One of the most crucial mechanisms to achieve this phenotype is activating the bone resorptive osteoclasts. As previously discussed, TDEVs’ cargo includes miRs that can have an extensive range of effects on the microenvironment. One study showed that breast cancer cells upregulate the production of miR-21, an upregulator of nuclear factor of activated T cell 1 (NFATc1). This cytokine strongly drives osteoclast survival and differentiation [32,185]. miR-21 achieves this by inhibiting the production of programmed cell death protein 4 (PDCD4), which is a suppressor of NFATc1 [32,156]. Another miR that has been shown to inhibit bone formation and induce osteoclastogenesis is miR-214 [157,158]. This miR was isolated in lung adenocarcinoma TDEVs [148]. It targets the pro-osteoblast differentiation transcription factor ATF4 to inhibit bone formation, as well as downregulate the expression of the *PTEN* gene to activate the PI3K/Akt/NFATc1 pathway and promote osteoclast survival and differentiation to induce bone resorption [148,157,158,159]. Another major osteoclastogenesis pathway that cancer cells target is the RANKL/RANK pathway. Some cancers, like prostate cancer, secrete EVs with CUB-domain containing protein 1 (CDCP1), a transmembrane protein inducer of osteoclastogenesis that has been shown to have an amplified effect in the presence of RANKL [160]. Other cancers, like breast cancer, secrete EVs that contain mRNA coding for RANKL, which are then taken up and translated by osteoblasts, thus increasing osteoclastogenesis [161]. Lastly, multiple myelomas cause osteolytic lesions by secreting various factors that activate RANKL, including CCL3 and MALAT1 [162,163].

The distinction between EV-packaged RANKL mRNA derived from breast cancer cells and the membrane-bound RANKL expressed by osteoblasts lies in both their mode of delivery and the subsequent modes of action they trigger in target cells. In the case of breast cancer, EVs serve as vehicles that encapsulate RANKL mRNA. Once delivered to recipient cells in the bone microenvironment, this mRNA is internalized and translated into the RANKL protein. This indirect signaling mechanism is distinct from the direct cell–cell interactions mediated by osteoblasts, where membrane-bound RANKL is preassembled at the cell surface and immediately available to engage its cognate receptor, RANK, on osteoclastic precursors [186,187,188].

Membrane-bound RANKL on osteoblasts has been extensively characterized to play a critical role in osteoclastogenesis by activating osteoclast differentiation via direct contact with RANK on osteoclast precursors. Moreover, membrane-bound RANKL participates in reverse signaling, wherein signals transmitted back into the osteoblasts contribute to the regulation of osteoblast differentiation and function [188,189]. This spatially restricted and tightly regulated mode of action ensures that osteoclastogenic signals are delivered in a high-fidelity and context-dependent manner [186,187]. In contrast, EV-delivered RANKL mRNA from breast cancer represents a distantly acting, paracrine mechanism that may facilitate osteoclast formation over a broader spatial range within the bone microenvironment. This EV-mediated horizontal transfer not only bypasses the requirement for direct cell–cell contact but also allows the tumor cells to modulate the recipient cells in a more distributed and potentially sustained fashion [190,191].

Mechanistically, membrane-bound RANKL is dependent on oligomerization and cell adhesion-dependent signaling to efficiently trigger osteoclast differentiation. It has been shown that such a configuration is more potent than soluble forms of RANKL [186]. By extension, EV-derived mRNA must first be translated into protein—an additional step that can affect the kinetics, localization, and ultimately the functional potency of the RANKL signal [192]. Furthermore, the translation of EV-packaged mRNA in target cells might lead to aberrant or ectopic expression of RANKL. This dysregulated expression has the potential to perturb the local bone remodeling balance, favoring osteolytic processes that are commonly associated with breast cancer bone metastases [191]. In contrast, the membrane-bound RANKL of osteoblasts is embedded in a well-regulated signaling unit that is integrated into the normal physiology of bone homeostasis [186,189].

While osteoblast-derived membrane-bound RANKL functions via direct contact and rapid reverse signaling to regulate both osteoclast and osteoblast activity in a highly controlled manner, EV-packaged RANKL mRNA from breast cancer initiates a more indirect, paracrine response. This involves translation in target cells leading to the production of RANKL protein that may act in a less spatially restricted manner, thereby contributing to a microenvironment favorable for osteoclastogenesis and bone degradation. Such mechanistic differences underscore the divergent roles these two forms of RANKL play in bone metabolism and cancer-mediated bone remodeling [186,187,188,189,190,192].

### 4.2. Osteoblast Suppression or Pathological Activation

Many interactions between primary cancers and osteoblasts have been studied. Many miRs found as cargo within TDEVs have been shown to inhibit osteoblastic function. These include miR-26a-5p, miR-27a-3p, and miR-30e-5p, which were all found in EVs released by prostate cancer cells [164]. Studies on EVs secreted from multiple myelomas isolated miR-129-5p and miR-103a-3p, impairing osteoblast differentiation and function [193,194,195]. All of these miRs exert their effects by inhibiting the Wnt or BMP signaling pathways [162,164,165]. Conversely, other miRs, like prostate cancer-derived miR-375 and miR-18a-5p, promote osteoblast differentiation and function by boosting the Wnt signaling pathway [196,197]. Lastly, for miRs, miR-141-3p has been isolated in EVs secreted by prostate cancer and, in one study was shown to promote osteoblast activity by inhibiting DLC1, an inhibitor of the p38MAPK pathway whose activation increases the expression of osteoprotegerin (OPG) in relation to RANKL, leading to more osteoblast activity [167]. On the other hand, another study concluded that this miR inhibits mesenchymal stem cell differentiation to osteoblasts via blocking the Wnt signaling pathway [168]. Further studies may be required to understand the true effect of this miR on osteoblasts. Other than miR, TDEVs of different cancers have been shown to contain TGF-β, or mRNA that codes for it, which functions to inhibit bone formation by osteoblasts [159,161,198]. Breast cancer TDEVs also reduced the expression Runx2, Osterix, and Collagen 1A1 in osteoblasts, which are essential regulators of osteoblast differentiation [199]. Another molecule shown to mitigate these factors’ levels is Dickkopf-1, which has been isolated in TDEVs of multiple myelomas [166,200,201]. Multiple myeloma TDEVs have also been shown to induce the release of IL-6, a downregulator of Runx2 and Osterix, from bone marrow stromal cells by activating the apyrimidic endonuclease 1 (APE1) and NFκB pathway [202].

## 5. Immune and Stromal Modulation by Tumor EVs

### 5.1. Macrophage Polarization and Myeloid Expansion

Tumor-derived extracellular vesicles (TDEVs) have also been shown to affect immune cells within the PMN, inducing an anti-inflammatory environment that supports their growth. One of the main ways TDEVs achieve this is by promoting the differentiation of macrophages to the anti-inflammatory M2 macrophages, also known as tumor-associated macrophages (TAMs). Some cancers, like lung adenocarcinoma, secrete miR-19b-3p within their TDEVs, which promotes M2 polarization of macrophages [169]. In a more roundabout way, it was found that some squamous cell carcinomas of the neck secrete EVs that are devoid of miR-34a, which triggers adrenergic innervation to the area [203]. This is important because the catecholamines released from these nerves trigger the differentiation of macrophages in the microenvironment to M2 macrophages [204,205]. Other than miRs, some cancers, like pancreatic cancers, secrete EVs that contain a high amount of arachidonic acid, which can be converted in the microenvironment into prostaglandin E2, which in turn promotes the differentiation of many anti-inflammatory cells, like M2 macrophages [171]. M2 macrophages promote tumor cell migration by expressing certain markers like arginase-1, TGF-β, and IL-10 [206,207]. TDEVs can also trigger the expansion and recruitment of myeloid-derived suppressor cells [208]. While the exact mechanism behind this process is unclear, it is theorized that it is mediated by the activation of STAT3 in a TLR2-dependent manner [209,210].

### 5.2. T, B, NK, and Dendritic Cell Dysfunction

Some tumors, like melanoma, secrete EVs that contain programmed cell death 1 ligand 1 (PD-L1) [211,212,213]. This molecule interacts with the programmed cell death 1 receptors on immune cells like T and NK cells, inducing them to undergo apoptosis [172,173]. This is especially vital for cancer survival as these cells are pivotal for the anti-tumor immune response [214]. TDEVs may also contain Fas ligand (FasL), an immunosuppressive molecule that interacts with Fas on CD8+ T cells, inducing their apoptosis [174,175,215]. T cell suppression is also a result of TGF-β, which was previously discussed as a component of TDEVs of various cancers [150]. Different cancer types significantly express CD73 in TDEVs [216]. This enzyme, 5′-nucleotidase, converts AMP into adenosine [217]. Adenosine promotes tumor growth by limiting T cell activity via adenosine receptor (P1R) signaling [176]. Dendritic cells are another immune cell that cancers target to promote immunosuppression. This is mediated by S100A8 and S100A9-rich TDEVs [177,218]. These factors combine to cause significant suppression of the anti-tumor immune response and allow tumor cells to grow and proliferate.

### 5.3. Non-Hematopoietic Stromal Targets

As previously mentioned, TDEVs play a role in inducing angiogenesis. EVs secreted by ovarian cancers have been shown to contain miR-205, which is internalized by endothelial cells to promote angiogenesis strongly [153]. Various cancers secrete phosphoglycerate mutase 1 (PGAM1) within EVs [219,220,221]. This is a key enzyme in aerobic glycolysis and has been associated with cancer progression and improved angiogenic and proliferative capacity of endothelial cells [154,155].

TDEVs also induce changes in adipocytes within the PMN. These adipocytes exhibit a phenotype characterized by reduced lipid content and markers, and are termed cancer-associated adipocytes (CAAs) [222]. Many factors, including TDEV-associated miR-126 and miR-144, mediate this change to CAAs [178]. TDEV-associated miR-155 may also promote lipolysis in adipocytes and beige/brown differentiation of adipocytes, which is associated with poor prognosis and survival [179].

TDEVs may also contain neurotrophic cargo. As previously mentioned, miR-34a-3p deficient TDEVs promote adrenergic axonogenesis. Other cancers may secrete EVs that contain brain-derived neurotrophic factor (BDNF) and nerve growth factor (NGF) [181,182,223]. These factors promote densely innervated tumors with poor outcomes. Some cancers induce neurite outgrowth by secreting axonal guidance molecules within TDEVs like EphrinB1 [183,224].

## 6. Experimental and Clinical Evidence Landscape

### 6.1. Model Systems

Three-dimensional bone-mimetic scaffolds integrated with fluorescent EV reporters offer a relevant method to analyze extracellular vesicles (EV) [225]. An example of one of these 3D bone-mimetic scaffolds is Ti64IA4V, a coated biometric scaffold allowing real-time description of EV production and release, as well as the added benefit of supporting osteogenic differentiation. A deeper understanding of EV-mediated signaling related to the bones is possible by augmenting the bone’s architectural and biochemical properties. Furthermore, the use of these platforms has the potential to be applied to pro-regenerative EVs as their production can be enhanced and used as an acellular tool for bone repair [225].

Microfluidic bone-on-chip systems are another model system that simulates bone microenvironments, allowing high-resolution tracking of intracellular communication and EV transport. One study [226] used human bone marrow physiology to study hematopoiesis and EV signaling simulated on a marrow-on-chip platform. Another study focused on exploring the role of EV trafficking in cancer metastasis by osteoblasts using a bone-on-chip model. This study concludes that potential exists for diagnosing and treating osteosarcoma using microfluidic detection of plasma EV membrane [227].

In model systems, organoids are three-dimensional cell clusters grown from stem cells using specialized 3D culture techniques. These techniques allow the cells to self-organize and regenerate, mimicking the structure and function of the original tissue [228]. EVs derived from organoids (OEVs) provide advantages such as increased yield of EVs, cargo consistency, and biocompatibility, which can be implemented for both diagnostic and therapeutic applications of EVs [229]. Fluorescent reporters observed in glioblastoma models allowed better analysis and real-time imaging [229].

### 6.2. Imaging and Quantification Technologies

Multiple imaging and quantification technologies can be used to study EVs. Intravital two-photon microscopy, super-resolution EV tracking, and single-EV Raman spectroscopy are among them.

Intravital confocal microscopy is combined with two-photon microscopy and fluorescence to investigate biological processes at the cellular level in living organisms. This technique is utilized in neuroscience, cancer, and immunology research [230]. The strengths of this technique include dynamic imaging of live cells and tissue, high localized excitation while reducing scattering, deep tissue imaging, reduced photodamage, initiation of well-localized photochemistry, adaptability to confocal microscopes, and the availability of automated alignment and laser tuning for non-specialized labs [231]. This technique, however, does not improve spatial resolution and causes more photodamage in thin samples [231].

An alternate method, super-resolution EV tracking, is a technique that allows reaching a lateral resolution of tens of nanometers, which enables nanometric biological structure visualization, with commercially available and open-access tools [232].

Single-molecule localization microscopy (SMLM) is a specific technique of super-resolution EV tracking. SMLM works by imaging random subsets of fluorophores over time, which are spatially and temporally confined; it relies on successive, not simultaneous, observation. It also requires fluorophores in a “dark state,” which is non-emitting, and “activated” emitting fluorophores. There are three subtypes of SMLM: stochastic optical reconstruction microscopy (STORM/dSTORM), PALM, and DNA PAINT. All three are based on the principle of localization with isotype switching; however, they differ in their probes as well as acquisition time; STORM uses photoswitchable dyes and blinking fluorophores and takes a long time to acquire, PALM uses photoactivatable fluorescent proteins and also takes a long time to develop, and DNA PAINT uses fluorescent DNA probes and takes a very long time to acquire [232].

STimulated Emission Depletion (STED) and Super-Resolution Structured Illumination Microscopy (SR-SIM) are two other types of SMLM. STED uses an excitation and redshifted depletion layer to confine fluorescence to an excitation area. The whole sample is then scanned to construct a high-resolution image. This technique’s advantages include ease of use and adding to confocal microscopes, fast acquisition, and image reconstruction time. The working principle behind SR-SIM is that it improves the sample resolution by illuminating it with patterned light to shift information into the detectable range. Fourier transform techniques are then used to combine multiple images from different pattern phases and orientations to reconstruct the image. The advantages of SR-SIM include the ease of application to specimens prepared for conventional methods. Fluorescence microscopy offers the ability to apply most available fluorophores, high resistance to photobleaching, and the ability to use multiple fluorophores simultaneously. The drawback is that the resolution gain is lower when compared to SMLM [232].

Along with intravital two-photon microscopy and super-resolution EV tracking, single EV Raman spectroscopy is another imaging and quantification technique that can use structural fingerprints to identify biochemical substances [233]. This method is advantageous as the data is flexible, high quality, classified by action state, and does not misclassify between cancer and control-derived particles [234]. However, the learning process weighting can lead to errors, which is why the intensity vector with the actual Raman features is more critical in reconstruction [234].

### 6.3. Clinical Correlates and Biomarkers

Many cancer biomarkers are associated with EV load. Prostate-specific antigen (PSA) is a key marker for prostate cancer diagnosis. In one study, EV load was found to have a strong correlation with PSA levels [235]. Because of this, EV quantification can be used as a tool for clinical workflow. This is further supported by the fact that the correlation does not just apply to highly sensitive testing kits, but also commercially available PSA testing kits, further showing the compatibility with workflows [236].

Besides PSA for prostate cancer, bone turnover markers such as β-CTX, PINP, PTH, osteocalcin, bone ALP, and TRAP5b differ as they are not fully accurate when evaluating bone reabsorption and formation [88]. Circulating EVs still provide benefits, as they can provide information about transcriptomic and epigenetic modification, allowing the early detection of downstream signaling pathway changes and traditional protein biomarkers [88].

Microfluidic Capture platforms allow efficient EV isolation from small sample volumes. Vn96 is a synthetic peptide with an affinity for the heat shock proteins (HSPs) found on EV surfaces. This peptide is used in a capture device for EVs [237]. The isolation process is efficient, as antibodies do not need to capture EVs [237]. Furthermore, Gold nano-islands are incorporated to expose localized surface plasmon resonance (LSPR), which detects the captured EVs [237]. Droplet digital PCR (ddPCR) showed the downstream genetic analysis capability of the capture device, and the integrity of the isolated vesicles was confirmed by atomic force microscopy. Because the process was completed in less than 30 min, it has the potential to be used in point-of-care diagnosis [237].

## 7. Therapeutic and Diagnostic Opportunities

### 7.1. Inhibiting EV Biogenesis, Release, or Uptake

Since EVs are central to cancer progression, many studies have explored ways to inhibit their biogenesis. Neutral sphingomyelinase 2 (nSMase2) is an enzyme involved in EV and ceramide biogenesis [238]. As a result, inhibiting this enzyme is a potential therapeutic target. An example of such an inhibitor is GW4869, which, in prostate cancer, has been shown to prevent EV release [239]. GW4869 inhibits EV-induced M2 macrophage polarization, which is associated with tumor progression. Experiments conducted in vivo show that treatment using GW4869 reduced tumor growth significantly [239].

RAB27A is a gene that is involved in vesicle trafficking. The inhibition of RAB27A has been studied in cancer progression. Rab27A and Rab27B inhibition have been shown to reduce EV release in HeLa studies and other cancer models [240]. Moreover, Rab27A plays a role in determining multivesicular body size, whereas Rab27B influences their localization [240]. Rab27A suppression has been shown to reduce tumor growth and metastasis in melanoma and breast cancer models, possibly due to a decrease in bone marrow recruitment. Bladder cancer models show that both Rab27A and B suppression cause the accumulation of tumor suppressive miRNA. Based on this, Rab27A suppression can potentially become a therapeutic target for EV-related cancer [240].

Integrin αvβ3 antagonists are agents that enhance tumor regression by inducing apoptosis of angiogenic blood vessels [241]. They function by affecting small-EV (sEV) adhesion and uptake from tumors [79]. One study demonstrated decreased CD63 expression in breast cancer cells after treatment with an αvβ3 antagonist (DisBa-01), suggesting a change in the sEV biogenesis and content. This shows that tumor microenvironment communication with sEVs is affected by αvβ3 antagonists and can serve as a target to inhibit cancer progression [79].

### 7.2. Extracorporeal or In Situ EV Depletion

Aethlon Medical’s Hemopurifier is an extracorporeal device designed to remove EVs originating from tumors. Pembrolizumab was used with the Hemopurifier in advanced head and neck squamous cell carcinoma in a phase 1 clinical trial (NCT04453046) [242]. Circulating EVs were reduced, enhancing immunotherapy efficacy by suppressing the EV immune response and restoring immune responses against the tumor [243].

### 7.3. Engineered EVs for Targeted Drug/Gene Delivery

Apoptotic bodies (ApoEVs) derived from bone marrow mesenchymal stem cells (BMSCs) are nanoscopic EVs with low immunogenicity and high biocompatibility, which can promote bone regeneration. One study developed ApoEVs conjugated to a bone-targeting peptide (Asp-Ser-Ser) using carbodiimide chemistry (DSPE-PEG-COOH as the linker) and was analyzed using confocal laser scanning microscopy. RING finger protein 146 (RNF146) was loaded into BMSCs by adenovirus transduction. The effects were studied in vivo and in vitro, which showed that osteogenesis was promoted [244]. Other than this study, there are multiple phase I trials currently running that aim to investigate the potential for the use of EVs in cancer therapy [245].

EVs have also shown great potential in gene editing. The delivery of CRISPR Cas9 RNP complexes within EVs traffics the RNP directly without transcribing and translating DNA in target cells, making this technique an efficient gene editing technique [246]. Various strategies have been developed to optimize the RNP loading, including fusion protein synthesis, post-translational modification, viral glycoproteins (VSV-G), and incubation without transfection reagents [246]. Diseases such as Hb Barts (β-thalassemia), Duchenne muscular dystrophy (DMD), and severe combined immunodeficiency (IL2RG) have applications for these techniques as gene therapies. EVs are advantageous to other delivery platforms as they are less immunogenic, more biocompatible, and efficient [246].

### 7.4. Liquid-Biopsy and Companion Diagnostics

Amid growing interest in EVs, technical standardization is vital because many methodologies have been used to isolate and analyze EVs. Following isolation, a variety of techniques have been employed to purify RNA. The influence of these disparate techniques on the results of downstream extracellular RNA (exRNA) sequencing and profiling remains unclear, raising the need to define “best practices” and eventual standardization. As exRNA diagnostic platforms become available, there will be requirements for clinical certification and manufacturing standards development. Furthermore, pressure will emerge to define and centralize biofluids from normative biological controls [247]. SNRPG, OST4, TOMM7, and NOP10 are stable and universally present EV-associated reference genes (RGs). One study suggested that these RGs can be used to enable the implementation and normalization of RT-qPCR to analyze EV-associated RNA cargo for research or clinical applications [248].

### 7.5. Regulatory and Manufacturing Challenges

GMP-compliant manufacturing of cell-derived vesicles (CDVs) will facilitate the preclinical and clinical evaluation of these emerging therapeutics in anti-inflammatory or regenerative medicine. This study also represents a crucial step in the development of this novel drug delivery platform based on CDVs [249].

One of the regulatory and manufacturing challenges in EV research and production is reducing batch-to-batch variability. Flow cytometry analysis (multiplex bead-based) was established and validated; 37 surface markers were incorporated along with two capture beads coated with control antibodies, which were shown to be reproducible [250]. Batches were considered indistinguishable statistically from previous batches if they had a Spearman correlation coefficient ≥ 0.9 and *p*  <  0.05 [250]. Another challenge with EV research and production is potency assay development. Parameters that determine clinical efficacy, such as dosing as well as delivery route, need to be considered; however, they are only fully evaluated in clinical trials [251]. Before testing in a clinical setting, standardized quantitative assays in lab-based experiments need to be conducted, which assist in predicting the therapeutic potential and fall within the guidelines under the International Council for Harmonization of Technical Requirements for Pharmaceuticals for Human Use (ICH) [251].

Currently, no United States Food and Drug Administration (FDA)-approved therapeutic EV products exist. The FDA sets CMC (chemistry, manufacturing, and control) requirements, and also guide developers to ensure the safety, identity, potency, and quality of EV products, including methods of ensuring reduced batch-to-batch variability and reliability in therapeutics, such as descriptions of production processes, analytic validation, and characterization [252].

The European Medical Agency (EMA) also has guidelines for requirements for developing EV-based treatments, which fall under the category of advanced medicinal therapy products (ATMPs). The guidelines are similar to those of the FDA, as they highlight the manufacturing and quality control processes that ensure the safety, identity, potency, and quality of EV products [253]. Guidelines from the FDA and EMA show how exhaustive the quality assurance process is to support safe and efficacious EV therapeutic products.

## 8. Methodological and Reporting Considerations

The Minimal Information for Studies of Extracellular Vesicles (MISEV) 2023 checklist, which is issued by the International Society for Extracellular Vesicles (ISEV), is a tool used to standardize research about EV, which thereby enhances the field of EV research by providing a framework for researchers to ensure best practice with regard to conducting EV and reporting data and results. Section 2, Section 3, Section 4 and Section 5 are mandatory and essential sections which cover nomenclature, collection and pre-processing, EV separation and concentration, and EV characterization, respectively [254].

Section 6, Section 7, Section 8 and Section 9 suggest, but do not mandate, best practices in EV research. Section 6 emphasizes reporting for EV characterization using various techniques like flow cytometry and proteomics. Section 7 advises on documenting EV release and cell interaction studies, including effects on cell biology, experimental conditions, and EV dosing relevance. It also recommends evaluating specificity and normalization in EV modulation [254].

Section 8 advises using appropriate negative controls, evaluating storage and preparation effects, justifying normalization methods, and conducting kinetic and dose–response studies. Section 9 highlights in vivo study considerations, including potential off-target effects of EV-blocking strategies, differences between endogenous and introduced EVs, and the importance of transparent reporting on labeling, detection, and administration parameters [254].

Compliance with these sections allows peer validation and comparison across studies, which enhances the study foundation. When the MISEV 2023 guidelines are followed, the field advances due to ensuring that findings align with the EV community’s expectations, as well as being methodologically reasonable. Researchers investigating EVs should refer to the official MISEV 2023 guidelines for these reasons [254].

Reproducibility, as well as collaboration, is essential in EV research and requires transparent data sharing. Platforms such as “EV Track” and “Vesiclepedia” serve as databases, facilitating transparent data sharing in EV research. EV TRACK (Transparent Reporting and Centralizing Knowledge) is a community-based database that emphasizes reporting experiments in a standardized fashion. Furthermore, interpreting and replicating studies is also facilitated [255]. Vesiclepedia is another database that compiles data (proteins, DNA, mRNA, miRNA, lipids, and more) from published and unpublished studies [256]. Data is sourced from over 3400 studies, with over 566,000 protein entries (http://microvesicles.org/). Both databases make data transparent and accessible, allowing studies to be reproduced and the field of EV research to be enhanced.

## 9. Knowledge Gaps and Future Directions

### 9.1. Single EV Multi-Omics to Resolve Heterogeneity

Single-EV multi-omics is an advanced analytical approach that simultaneously profiles individual extracellular vesicles (EVs) across multiple molecular layers—including proteins, RNAs, DNAs, and lipids. This powerful technique uncovers the heterogeneity and functional specialization of EV subpopulations, revealing how specific EV types contribute to metastatic niche formation and serve as early diagnostic indicators in various disease contexts [257,258]

A key technological advancement is the development of seiSEQ, a highly sensitive and scalable DNA-barcoded immunosequencing platform. This droplet-based microfluidic system labels EVs with DNA-barcoded antibodies, encapsulates them alongside barcoded beads, and sequences amplicons to resolve the protein composition of each vesicle. seiSEQ enables ultra-high-throughput single-EV protein profiling with virtually unlimited multiplexing capacity. Beyond EVs, it is also applicable to viral, bacterial, and synthetic nanoparticles, positioning it as a promising tool for next-generation diagnostics and biomarker discovery [259].

In parallel, other innovations such as SPIRFISH (Single-Particle Interferometric Reflectance Imaging Sensor) have improved resolution by enabling the simultaneous detection of surface proteins and internal RNAs within single vesicles. SPIRFISH, for example, can distinguish infectious HIV-1 virions from contaminating EVs by confirming both viral proteins and genomic RNA in the same particle. Such capabilities are particularly valuable in bone metastasis research, where identifying bone-tropic EV subpopulations offers mechanistic insights and supports precise biomarker discovery [260].

Single-EV multi-omics is most powerful when integrated with complementary platforms that broaden the scope of analysis:

Nano-flow cytometry (nFCM) enables the rapid, high-throughput characterization of thousands of EVs per minute, based on size, concentration, and surface marker expression [261].

Mass spectrometry (MS) supports in-depth proteomic and lipidomic profiling by quantifying a broad range of biomolecules within individual vesicles [262].

Next-generation sequencing (NGS) analyzes EV-associated nucleic acids (e.g., miRNAs and mRNAs), offering insights into transcriptomic and genetic content.

In bone metastasis, NGS-based EV profiling has helped identify biomarkers and therapeutic targets specific to metastatic lesions [263]. In other cancers, such as non-small cell lung cancer (NSCLC), exosomal miRNA panels specific to adenocarcinoma and squamous cell carcinoma have shown high diagnostic accuracy, reinforcing the clinical utility of NGS for noninvasive diagnostics [264].

In the context of bone metastasis, single-EV multi-omics has uncovered distinct EV subtypes associated with specific tumor cell states and their capacity to colonize bone. This approach has deepened our understanding of how EVs mediate tumor progression and interact with the bone microenvironment [13]. It also reveals novel biomarkers and functional cargo involved in pre-metastatic niche formation and disease dissemination. From a clinical perspective, single-EV multi-omics holds significant potential for early detection, prognostic evaluation, therapeutic monitoring, and personalized treatment of bone metastases [103,265].

Despite its promise, single-EV multi-omics faces significant technical and practical challenges. These include low throughput, difficulty detecting low-abundance molecules, and a lack of standardization that hampers reproducibility. Integrating multi-omic datasets also remains complex, requiring advanced computational tools and bioinformatics expertise [258,266]. High costs, regulatory barriers, and the need for large-scale validation studies currently limit translation into routine clinical use. Continued innovation in methodology, computational frameworks, and infrastructure will be essential to realize its potential in diagnostics and precision medicine fully [267].

### 9.2. Triggers of Selective Cargo Loading in Hypoxic, Acidic Bone Niches

Another field that requires more investigation is selective EV cargo loading triggers. The bone metastatic niche is characterized by hypoxia and acidity, driven by increased tumor metabolic activity, poor vascularization, and heightened glycolysis. These conditions selectively influence the EV cargo, enhancing the loading of bioactive molecules tailored to the tumor microenvironment [268]. Despite these findings, the precise mechanisms by which hypoxic and acidic stress influence EV biogenesis and cargo selection remain not fully understood [269]. Identifying the molecular pathways involved, particularly within the bone microenvironment, may provide valuable insights for targeted therapeutic strategies to manipulate EV signaling in bone metastasis [270].

### 9.3. Sex- and Age-Specific EV Effects on Bone Cells

Biological sex and aging significantly influence EV content, concentration, and function, potentially impacting the bone metastatic microenvironment [271]. While sex-based differences in bone biology and immune regulation are well established, studies exploring how these variations manifest in EV-mediated signaling remain limited. Nonetheless, evidence suggests that EV cargo differs between sexes, potentially affecting interactions with osteoclasts and osteoblasts in the bone niche [272].

Aging is associated with substantial alterations in EV secretion and cargo composition, particularly involving microRNAs linked to senescence. For instance, aged muscle-derived EVs are enriched in miR-34a-5p, which impairs bone marrow stem cell populations, suggesting that skeletal muscle may serve as a source of senescence-associated EVs that disrupt bone homeostasis [273].

Despite age-related declines in EV functionality, EVs from younger plasma sources have demonstrated the capacity to reverse aging-associated phenotypes in preclinical models, enhancing mitochondrial function and promoting tissue regeneration [274,275]. Additionally, youthful EVs increase PGC-1α expression, counteracting mitochondrial deficiencies in aged tissues [275]. These findings underscore the potential for EV-based therapies to restore bone homeostasis in aging populations.

Despite emerging insights, direct evidence linking sex- and age-specific EV variations to bone metastasis remains limited. Most studies lack stratification by age and sex, resulting in generalized conclusions that fail to capture patient-specific EV profiles [271]. Furthermore, while genetic and age-related factors influence EV formation, the regulatory mechanisms governing EV biogenesis and cargo selection remain poorly defined [276].

Future research should focus on developing age- and sex-specific models to delineate how EV content and function vary across different patient populations. Understanding these dynamics may facilitate the development of personalized EV-based diagnostics and targeted therapeutics for bone metastasis.

## 10. Conclusions

Extracellular vesicles (EVs) have rapidly transitioned from peripheral curiosities to central players in our understanding of how tumors colonize the bone. Far from passive carriers, EVs serve as strategic messengers, delivering molecular cargo that orchestrates critical steps in metastasis: guiding circulating tumor cells to specific skeletal sites, modulating the behavior of local stromal and immune populations, and reprogramming bone-resident cells, osteoclasts, osteoblasts, and osteocytes, from protectors into facilitators of disease. Recognizing this EV-driven command hierarchy adds a new dimension to long-standing models of bone metastasis, such as the “vicious cycle” of osteolysis, now seen through the lens of vesicle-mediated signaling.

Clinically, this paradigm shift holds significant promise. A simple liquid biopsy detecting EV-derived biomarkers could one day predict skeletal metastasis months before radiographic lesions emerge. In parallel, therapies that block EV biogenesis, release, or interaction with bone cells may serve as valuable adjuncts to existing anti-resorptive agents. However, the field remains in its early stages. Standardized protocols for EV isolation, high-resolution single-vesicle multi-omics to resolve functional heterogeneity, and rigorous clinical trials evaluating EV-targeted strategies are urgently needed. Still, the trajectory is clear: controlling these nanoscale messengers may unlock a new strategy to combat the formidable challenge of bone metastasis.

## Figures and Tables

**Table 1:** Bioactive cargos identified in tumor-derived extracellular vesicles and their cell-specific effects that shape the bone metastatic niche.

EV Cargo	Target Cell	Effect	Reference
TGF-β	Fibroblasts	Activates SMAD Transcription factors and SRF inducing their change to CAF	[149]
	T Cells	Suppresses CD8+ T cells by directly reducing proliferation and activating regulatory T cells	[150]
Sphingomyelin	Endothelial Cells	Promotes endothelial cell migration, tube formation, and neovascularization by interacting with S1P receptors on endothelial cells	[151]
CD147		Promotes endothelial cell migration and increases MMP production	[116]
miR-9		Promotes endothelial cell migration by activating JAK-STAT pathway	[117]
miR-210		Promotes tube formation, migration and proliferation by targeting EphrinA3	[152]
miR-135		Enhances angiogenesis by targeting factor-inhibiting HIF-1	[120]
Annexin A2		Assists in the conversion of plasminogen to plasmin	[125]
miR-105		Downregulates tight junctions and destroys the barrier function of endothelial monolayers by targeting Zonula Occludens 1	[121]
miR-181c		Contributes to actin degradation through the suppression of cofilin	[122]
miR-205		Induces angiogenesis via the PTEN-AKT pathway	[153]
PGAM1		A key enzyme in aerobic glycolysis that promotes endothelial cell proliferation	[154,155]
miR-21	Osteoclasts and Precursors	Promotes osteoclast survival and differentiation by upregulating NFATc1	[32,156]
miR-214		Inhibits osteoblast differentiation by targeting the ATF4 transcription factor and downregulates the expression of PTEN	[157,158,159]
CDCP1	Osteoblasts	Induces osteoclastogenesis by upregulating RANKL expression	[160]
RANKL mRNA		promotes osteoclastogenesis	[161]
CCL3		Induces RANKL expression	[162]
MALAT1		Induces RANKL expression	[163]
miR-26a-5p		Inhibits the Wnt and BMP signaling pathways	[162,164,165]
miR-27a-3p			
miR-30e-5p			
miR-129-5p			
miR-103a-3p			
Dickkopf-1		Reduces the expression of Runx2, Osterix, and Collagen 1A1 downregulating differentiation	[166]
miR-141-3p		Promotes osteoblast activity by inhibiting DLC1	[167]
	Mesenchymal Stem Cells	Blocks their differentiation to osteoblasts by blocking the Wnt signaling pathway	[168]
miR-19b-3p	Macrophages	Targets and inhibits PTPRD-mediated dephosphorylation of STAT3	[169]
miR-9-5p		Promotes conversion of cholesterol to 25-hydrocholesterol which induces differentiation to M2 macrophages	[170]
Arachidonic Acid		Converted to PGE2 to promote the differentiation to M2 macrophages	[171]
PD-L1	NK Cells	Induces apoptosis in immune cells	[172,173]
	T Cells		
FasL		Interacts with the Fas receptor to induce apoptosis	[174,175]
CD73		Converts AMP to adenosine which limits T cell activity via P1R signaling	[176]
S100A8 and S100A9	Dendritic Cells	Inhibit dendritic cells maturation and differentiation by decreasing the expression of CD83, CD86, IL-12, and IL-15	[177]
miR-126	Adipocytes	Disrupts the IRS/Glut-4 signaling pathways, activating AMPK/autophagy pathways, and stabilizing the expression of HIF-1α	[178]
miR-144		Mediates the beige/brown differentiation via the MAP3K8/ERK1/PPARγ axis	
miR-155		Suppresses adipogenesis and enhances brown adipose differentiation by targeting C/EPBβ	[179]
miR-34a	Sensory Nerves	EVs deficient in miR-34a are derived from cancer cells that lost *TP53* and promote adrenergic differentiation of nerves	[180]
BDNF and NGF	Neurons	Activates the BDNF/Tropomyosin receptor kinase B (TrkB) pathway which promotes neuron migration and invasion	[181,182]
EphrinB1		An axonal guidance molecule that induces neurite outgrowth	[183]

## Data Availability

No new data were created or analyzed in this study. Data sharing is not applicable to this article.
